# Fake news and real news: which is which?

**DOI:** 10.3325/cmj.2022.63.495

**Published:** 2022-10

**Authors:** Charles H. Calisher

It is almost humorous – that the so-called news emanating from national capitals, online social media sources, TV “talking heads,” and some other sources of public “information” cannot be relied upon with confidence. That is because certain individuals do not want the rest of us to know what is actually happening, what they are actually doing, or what they are scheming to do, if given half a chance. The list that follows is a combination of both real and fake news that I have either selected from daily collections or fabricated completely on my own. If you cannot tell the difference between them, you are not thinking logically, you are gullible, or you take political and other advice from the person who cuts your hair.

1. **Marion Anne Perrine (“Marine Le Pen”) defeated Emmanuel Macron in the recent second round of France's presidential election.** Macron has claimed that the election was stolen from him and has asked Donald Trump for advice on how to prove this to the French electorate, as Trump has done with some small success in the U.S.A. following his own loss. Trump has volunteered to move to Paris, build himself a hotel, and advise Macron from there. His family will wait for him in Washington, D.C. while they await their trials, sentencing, and hangings.

2. **All four remaining Nazis in Ukraine have been moved by Russia to Argentina, Chile, Bolivia, France, Paraguay, Brazil, or elsewhere to be with their surviving “buddies” so they can talk about the “good old days.”** Russia will soon remove its troops from Ukraine, after reducing it to being the largest corn, wheat, and mine field on earth. Popcorn will be planted in areas where mines were left by the Russian Army.

3. **Vladimir Vladimirovich Putin, having accumulated an estimated $200 billion dollars ( = an infinite number of rubles), has left Russia to live on Elba, Italy, to visit Napoleone di Buonaparte and ask him how he did it.** Putin does not know that Napoleon is dead and no one is brave enough to tell him. He leaves behind an impoverished Russia, which is purchasing corn from Ukraine. Many ordinary Russians are furious because most of the “enemy” killed in Ukraine were old women, and young children, and most of the infrastructure they demolished were hospitals, schools, nurseries, and pizza restaurants, all of which were considered threats to Russia. In return, the Ukrainians have devastated Russia, at least the parts that Putin’s policies have not already damaged.

4. **The world is flat, no matter what you have heard.**

5. **Mickey and Minnie Mouse have been infected with an as yet unidentified hantavirus**. It is assumed that they were infected in their residence but, irrespective of this epidemiological detail, the important point is that they have been infected with a newly recognized rodent-borne virus, whatever it is (suggested name, sight unseen: Cartoon virus, species *Disney orthohantavirus*). These rodents had been complaining for a few days that they did not feel completely healthy but hantaviruses usually cause very mild or asymptomatic infections in rodents, so their signs and symptoms simply might reflect “common colds.” Nonetheless, two pieces of useful information will burden the scientific record further when their species is finally determined and the specific hantavirus with which they have been infected is determined. Mr. and Mrs. Mouse (I assume they are married but I have never seen that in writing.) have been deluged with well wishes from a large number of children world-wide and by V. Putin, who denies any involvement in their infections.

6. **Former British parliamentarian Neil Parish** resigned his position in late March 2022 after female colleagues caught him watching pornography on his laptop in the House of Commons. Parish, also a farmer, has denied these charges and, in his defense, said that corn was the problem, that he was simply searching for a combine harvester when he accidentally stumbled upon a porn site while looking for a shiny new Dominator combine with a Tesla engine that allows the combine to hover. Meanwhile, Parish has secured an exclusive contract with the United Nations to plant corn in what was Ukraine.

7. **Russia is considering declaring war on Israel** but Russian soldiers have learned how to sabotage the tanks the Motherland sent with them to Ukraine, so the Russian Army will have to fly to Israel on El Al Airlines (Aeroflot is now serving only 13 countries, and ice hockey players wanting to go home, can fly only through Kazakhstan and Armenia) and pay extra money (in US dollars, by weight) to be accompanied by their equipment. El Al has arranged to subcontract these deliveries with unnamed airlines, which have agreed verbally to drop their cargo somewhere over the Mediterranean Sea.

On a related note, the Italian Government has impounded a $US 700 million mega yacht, “Scheherazade,” linked to some Russian oligarchs. Very sadly, many thousands of Russian conscripts have died in the most recent efforts to acquire Ukraine and many Russian tanks, trucks, ships, and bottles of vodka have been destroyed as well. Thus, if anyone had planned to leave Russia by mega yacht, they had better change their plans and learn to swim.

8. **Mexico wants Texas returned to Mexico.** People in the rest of the US states would likely have no objections to this but Texans are special and are willing to re-fight the Battle of the Alamo (1836), which they had lost a few times previously. These days, more Mexicans arrive daily into Texas than arrive in Mexico from Texas, the latter being mostly tourists and drug dealers going back to Mexico in preparation for their next return trip.

9. **England wants the US (colonies) back:** The Brits would rather have the US be part of England than have England be part of Europe. As the British speak strangely, have dreadful eating habits, and tolerate abysmal weather, only 47 people traveled to England last year from the US, whereas 416 000 Brits arrived in the US All the latter disappeared almost immediately and have not been located, except for those whose bodies were found in vehicles that had been driven on the left sides of the roads and those who had adopted the metric system.

Britain could have avoided many of their problems long ago. They could all have come over here by about 1750, abandoned all those islands to the Irish, easily learned a new and better, more flexible form of English, gotten rid of their unelected royalty, left all those foxes (*Vulpes* sp) to themselves and their natural predators, saved a fortune by not building huge cathedrals paid for by poor people, and eventually listened to Mohandas Gandhi, whose philosophy would have made the US a better place. [When asked what he thought of Western civilization, Gandhi said, “I think it would be a good idea.”]

10. **Russia wants Alaska back:** (https://www.msn.com/en-us/news/world/alaska-gov-sends-message-to-putin-allies-after-russia-demands-state-back/ar-AAZlJfO?rc=1&ocid=winp1taskbar&cvid=d50e1ed2ca5e416eda8c101fbe0d6da9n)

In 1867, the US paid good money for Alaska ($US 7.2 million; $US 140 million in today’s slightly inflated economy) but, nowadays, that would only be enough to pay the monthly rent for a building in downtown Juneau, Alaska’s capital. At the time of the sale, Russia wanted to sell its Alaska territory to the US because Alaska was remote and would have been difficult to defend, rather than risk losing it in battle with a rival such as the former Great Britain. We thought we were doing Russia a favor but then gold, other valuable minerals, salmon (fish of the family Salmonidae), and warm clothing were discovered there, so we probably will not return Alaska to Russia, if there is a “Russia” to return it to.

Russian politicians recently also have called for Russia to take back an area near San Francisco, California. In response, the Governor of Alaska said, “To the Russian politicians who believe they can take back Alaska: Good luck.”, although why he is responding at all is unclear. In addition, a local California official said, “It reminded me that America does not have a monopoly on crazy politicians. It's not even worth talking specifics, it's just noise,” she said. Anyway, if any Russians traveled to that area, tasted some of the local wines and foods, spent some days in San Francisco, watched a few baseball games, and otherwise enjoyed themselves there, they probably would not want to return to Russia. Attempted reacquisition is altogether a bad idea for everyone.

**11. Other disputes over who owns what:** If you want to have a good laugh, see https://en.wikipedia.org/wiki/List_of_territorial_disputes#Americas There are 30 on-going disputes between U.N. member/observer states in Africa; 42 in Asia; 22 in Europe; 9 in North America; 6 between Canada and the US; 9 in South America; 4 in Oceania; 68 in Asia involving states outside the U.N.; 20 disputes within the US between internal entities; 3 in Antarctica; and many others that have been settled when countries decided that all the arguing was not worth the energy and expense. In one dispute over an underwater volcano, a new piece of land was produced but later disappeared beneath those waters when the mountain was reclaimed by the sea. That did not discourage one of the disputants from planting its flag on top of that drowned mountain, under water, where it remains to this day, unless fish have laid claim to it in the meantime.

**12. Jorge Mario Bergoglio (a.k.a. “Pope Francis”), is the Bishop of Rome and therefore head of the Catholic Church.** Also, as sovereign of the Vatican City State, Pope Francis seems to have had enough of child molestation charges against officials of the Catholic Church and now has declared war on places where such people are found, which is about everywhere. For many years, the Church had quietly been accumulating weapons of war, ammunition, and an army of volunteers, and these have been sent to countries where the offenders live and where they tell others how to behave. Pope Francis has apologized in advance for these actions and explained the need for them as, “I have had just about enough of this crap.”

**13. U.N. calls for a renaming of “monkeypox virus.”** Objections have been raised against the name “monkeypox,” understandable in this age when people seem to be overly sensitive about names and pronouns. The main problem is that monkeys also are offended by the name, themselves calling it “humanpox virus.” The natural origin of this virus, as yet undetermined, possibly is a rodent-borne virus. Because it makes monkeys [and apes: Great ape 1, gorilla (*Gorilla* sp), Great Ape 2, chimpanzee (*Pan troglodytes*), Great Ape 3, orangutan (*Pongo* sp), Great Ape 4, bonobo (*Pan paniscus*), Great Ape 5, human (*Homo sapiens*)] ill, these vertebrates probably are not reservoirs of the virus, and its natural origin has not yet been established, so perhaps *Orthopox neglectovirus* might be an appropriate, if taxonomically horrible, name for this virus. If in the unlikely event that a dinosaur was the first vertebrate host, we likely are in for a long haul but research funding also will last a long time.

The point of all this is that it may be difficult to distinguish between news that is factual and news that may be manufactured, ie, not true at all. The danger is that if reported news is actually misinformation it is propaganda, information that is biased or misleading, and likely used to promote or publicize a particular political cause or point of view. Those who take such reports as factual accounts of real events may find themselves instead manipulated by those who want to change opinions. My attempts here to humorously report nonsense as facts are intended to show, in a bizarre way, how actions that are partially or believably true are not true. We scientists rely on honest peer review in honest journals, not “fake” journals, of which there are many. In this very busy world, we all must stay on guard against such partial truths, such as the justifications being given for the current barbarous attack on Ukraine.

